# A new clinical test for sensorimotor function of the hand – development and preliminary validation

**DOI:** 10.1186/s12891-017-1764-1

**Published:** 2017-09-26

**Authors:** Ulrik Röijezon, Ragnar Faleij, Petros Karvelis, George Georgoulas, George Nikolakopoulos

**Affiliations:** 1 Department of Health Sciences, Luleå University of Technology, Luleå, Sweden; 2 Laboratory of Knowledge and Intelligent Computing, Department of Computer Engineering, Technological Educational Institute of Epirus, Arta, Greece; 3 Control Engineering Group Department of Computer Science, Electrical and Space Engineering Luleâ University of Technology, Luleâ, Sweden

**Keywords:** Hand, Image analysis, Laser pointer, Proprioception, Sensorimotor, Tracking task

## Abstract

**Background:**

Sensorimotor disturbances of the hand such as altered neuromuscular control and reduced proprioception have been reported for various musculoskeletal disorders. This can have major impact on daily activities such as dressing, cooking and manual work, especially when involving high demands on precision and therefore needs to be considered in the assessment and rehabilitation of hand disorders. There is however a lack of feasible and accurate objective methods for the assessment of movement behavior, including proprioception tests, of the hand in the clinic today. The objective of this observational cross- sectional study was to develop and conduct preliminary validation testing of a new method for clinical assessment of movement sense of the wrist using a laser pointer and an automatic scoring system of test results.

**Methods:**

Fifty physiotherapists performed a tracking task with a hand-held laser pointer by following a zig-zag pattern as accurately as possible. The task was performed with left and right hand in both left and right directions, with three trials for each hand movement. Each trial was video recorded and analysed with a specifically tailored image processing pipeline for automatic quantification of the test. The main outcome variable was Acuity, calculated as the percent of the time the laser dot was on the target line during the trial.

**Results:**

The results showed a significantly better Acuity for the dominant compared to non-dominant hand. Participants with right hand pain within the last 12 months had a significantly reduced acuity (*p* < 0.05), and although not significant there was also a similar trend for reduced Acuity also for participants with left hand pain. Furthermore, there was a clear negative correlation between Acuity and Speed indicating a speed-accuracy trade off commonly found in manual tasks. The repeatability of the test showed acceptable intra class correlation (ICC_2.1_) values (0.68-0.81) and standard error of measurement values ranging between 5.0–6.3 for Acuity.

**Conclusions:**

The initial results suggest that the test may be a valid and feasible test for assessment of the movement sense of the hand. Future research should include assessments on different patient groups and reliability evaluations over time and between testers.

## Background

Sensorimotor control is a term commonly used when referring to the nervous system’s transmission, processing and integration of sensory information (mainly visual, vestibular and somatosensory systems, including proprioception) and initiation and transmission of motor commands to the skeletal muscles [1, 2]. Proprioception can be defined as the conscious and unconscious sense of joint position and movement but also force; thereby including the three elements of position, movement, and force sense. Proprioception has multiple roles in sensorimotor control. These include updating the perception of the constitution and position of the body parts (body schema) and motor planning before movement, regulation of muscle stiffness, feedback and feed-forward control during movements, as well as predictions and evaluation of the result of motor commands after the movement which is important for motor learning [3]. Hand proprioception is, together with tactile information, also important for haptic awareness, i.e., awareness of shapes, sizes, weights and textures of objects [4]. Moreover a well-integrated proprioception with visual information is important for eye-head-hand coordination in many functional tasks and activities [5, 6].

The sensorimotor control of the hand is of immense importance for physical functioning and abilities such as gripping, lifting and manipulation of objects in the hand, but also for sensory information about objects and the immediate surroundings and for communication. Well adapted sensorimotor function of the hand is thereby important for daily activities, such as dressing, cooking and manual work. It is moreover requisite for individuals and professions with high demands on precise and well-coordinated fine motor control, e.g., musicians, illustrators/artists, surgeons, dentists, hair dressers but also for many athletes, for example those involved in racket or throwing sports.

Due to the diversity of functions of the hand, from precise movements and grips with small well adapted forces to fast ballistic movements, power grips and heavy lifting, proprioception is exceptionally important for the sensorimotor function of the hand. This is also anatomically and physiologically represented in the hand and fingers, which are highly condensed with mechanoreceptors contributing to proprioception, including muscle spindles, golgi tendon organs, mechanoreceptors in the joints, ligaments, fascia and skin as well as in the central nervous system (CNS) with large representations of the hand in the somatosensory and motor cortex [7].

Various musculoskeletal disorders, with or without traumatic onset, and neurological disorders can lead to disturbed sensorimotor functions of the hand. Common findings include altered neuromuscular coordination and disturbed somatosensory input and/or processing, including proprioception [8, 9]. Disturbed hand proprioception has been reported for musculoskeletal and neurological disorders such as fractures [10], arthritis [11], complex regional pain syndrome [12], sensory neuropathy [13], Parkinson’s disease [14] and stroke [8, 15]. Disturbed acuity in hand movements has also been reported in more proximal musculoskeletal disorders such as neck and arm pain [16, 17].

The importance of proprioception in musculoskeletal disorders has received increasing attention in recent decades in research and clinical work, including hand rehabilitation [18, 19]. However, feasible, affordable and accurate objective methods for assessment of proprioception are relatively scarce in the clinical setting. Clinical tests for assessment of wrist and finger proprioception often involves joint position sense measured with manual goniometer [20]. In movement science laboratories specific equipment are used for more accurate objective assessments of measures of position and movement sense, such as motor driven equipment and 3D motion capture systems [12, 21, 22]. These equipment are however not suitable for the general clinic due to cost and technological complexity involved. Graphonomic tests such as hand writing, drawing or tracking tasks with pen and paper techniques are valuable to evaluate sensorimotor function of the hand [23, 24]. These tests though involve movements with supported hand and/or pen and thereby also tactile input and movement adaptation due to the hand and pen contact with the surface. In this article, we suggest a feasible and affordable method for assessment of the movement sense of the wrist with unsupported freely moving hand by using a laser pointer and a novel software for swift and accurate objective evaluation of results. The objective of the study was to develop and conduct a primary evaluation of the validity of a clinical test with automatized scoring software for objective assessment of movement sense of the wrist.

## Methods

A new clinical test and automatic scoring tool for the assessment of movement sense of the wrist was developed and evaluated using an observational cross-sectional design. Ethical approval was achieved from The Regional Ethical Review Board in Umeå, Sweden (reference number 2016-71-31). Due to the large variation in dexterity demands among different occupations this study includes participants with the same profession, i.e., physiotherapist. This convenience sample of a non-patient group was included to gain normative data in a relatively homogenous group and evaluate differences due to handedness and variability over repeated test trials. Musculoskeletal disorders are however common in the general population, thus data was collected for any current or recent hand disorder since this was expected to affect the results. Data were collected at Luleå University of Technology in Luleå and at Haninge Physio Center in Stockholm by the same test leader during a 4 week period during spring 2016. Fifty participants were deemed suitable to investigate validity of the test which included, e.g., ability to reveal differences between dominant and non-dominant hand and repeatability over repeated trials. A previous study involving tracing task described as a digital equivalent of the pen and paper test showed significant better performance for the dominant hand in a group of 12 adults [25]. We expected fifty participants sufficient to assure statistical power to reveal any differences in handedness with our test. Moreover, fifty participants performing three trials has been suggested for reasonable precision of reliability estimates [26], including evaluation of ICC when expecting an ICC > 0.6 and aiming for a 95% CI width of ≤ 0.3 [27].

### Participants

Participants were recruited as a convenience sample at physiotherapist clinics and physiotherapy courses. All participants were currently working as a clinical physiotherapist. Inclusion criteria were physiotherapist by profession and currently working as a clinician. Exclusion criteria were a considerable reduced range of motion or neuromuscular dysfunction, e.g., due to injury or operation of hand or lower arm, that would preclude the participants ability to complete the task, visual impairment that could reduce the ability to visualize the target and the laser dot, or evidence of any neurological or rheumatic disease.

### Assessments

**Fig. 1:**
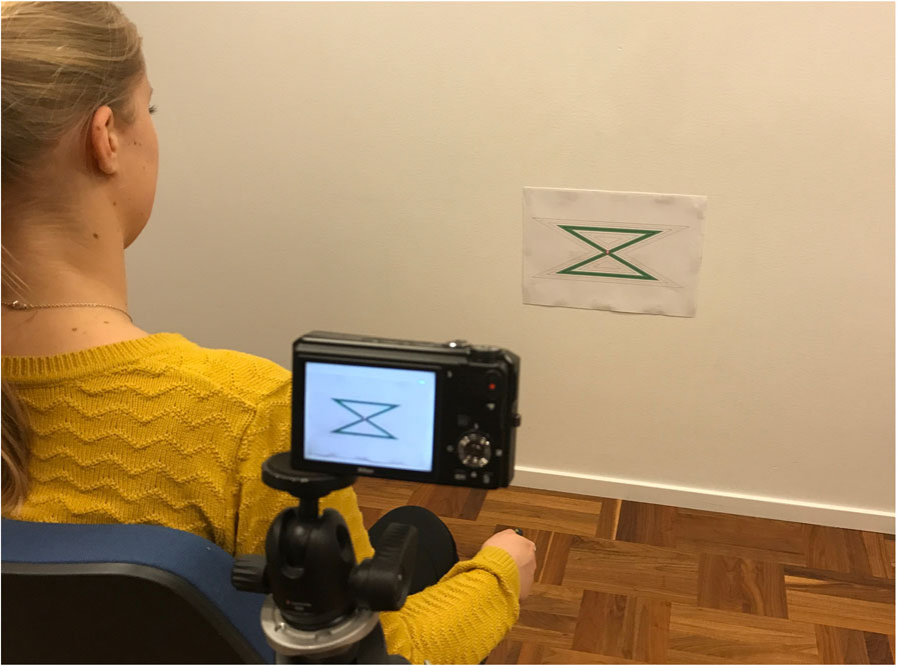
Illustration of the target pattern, camera position and the test position of the participant

The assessment task was to follow a thin black line at the center of a zig-zag pattern as accurately as possible with a hand held laser pointer (Fig. 1), performed with self-chosen speed. The black center line of the zig-zag pattern was 1 mm thick and made up a 1 m long trace. The target pattern was printed on an A3 paper board and attached to the wall. The task was performed with left and right hand, both in left and right directions, i.e., altogether four different hand movement tests. Three trials were performed for each hand and direction, i.e., 12 trials were performed by each participant. Each trial took approximately 15-45 s to complete.

The participant was seated on an office chair with lower arm resting on the chair’s armrest during the test. An erect seated position with spine in neutral position was instructed. Hips and knees were approximately 90°, wrist was placed just distal to the armrest allowing for free movement of the hand and the lower arm was positioned in neutral pronation-supination. Before initiating the task the zig-zag pattern was positioned horizontally with the center cross of the pattern at the place of the laser dot when pointing the laser with the wrist in neutral position, i.e., with slight ulnar deviation and dorsal extension. The distance between the laser pointer from Logitech Legamaster LX-1 (red laser) and the target was 1.00 m. A Nicon video camera, with a resolution of 1280 × 720 pixels and with a frame rate of 30fps and 24bits color depth, was placed in a fixed position on a tripod just behind and lateral to the shoulder of the hand being tested. The complete target pattern with the moving laser dot was filmed during each trial for evaluation of test results. Furthermore, a questionnaire was used to collect demographic data about the participants including gender, age, height, weight, handedness, current experience of hand dysfunction and hand pain within the last 12 months.

### Software for automatic scoring of outcome variables

**Fig. 2:**
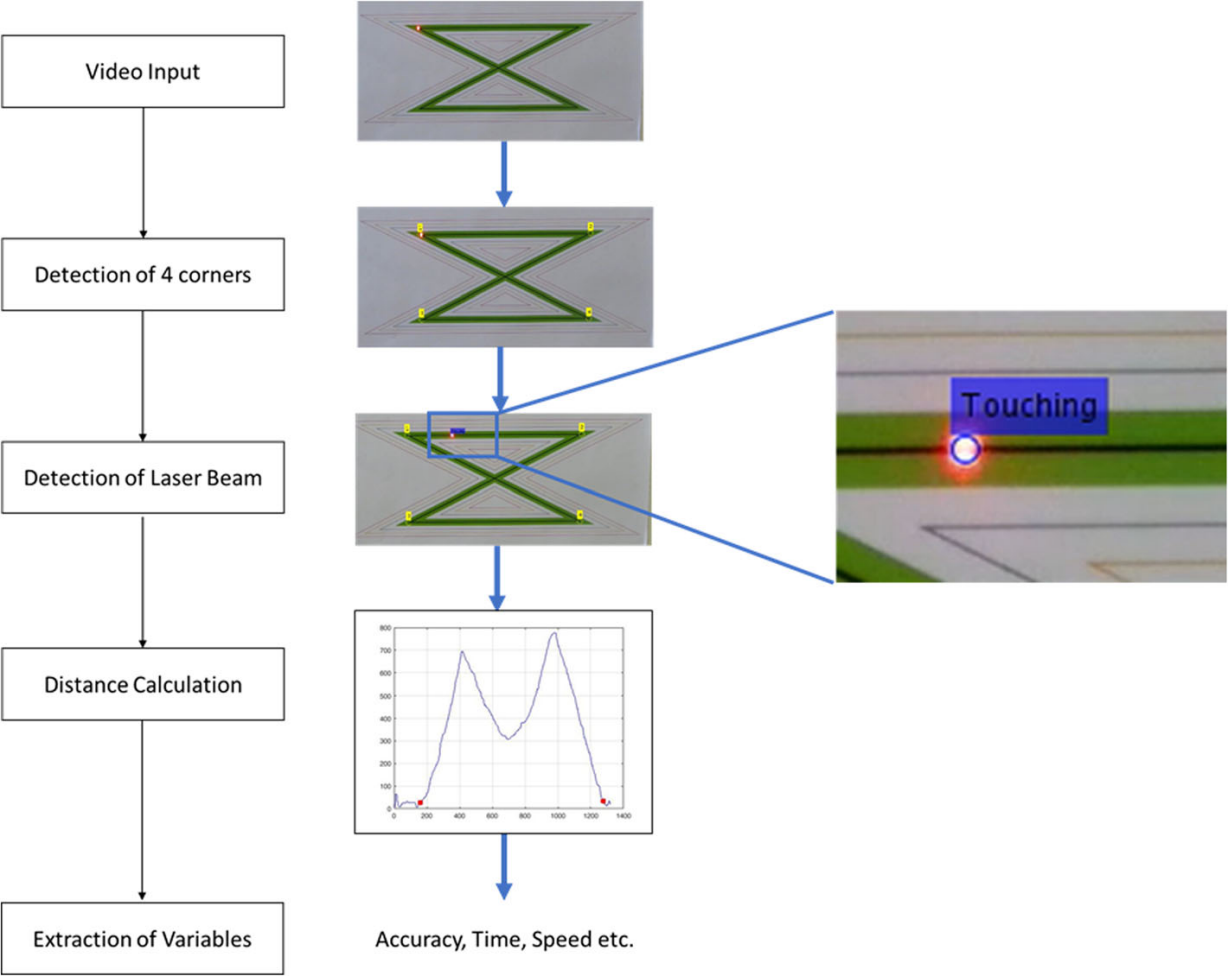
A flowchart of the software method with parallel indication of the image processing steps on the captured data and a highlighted view of the dot path

In order to track the laser dot, a MATLAB software program was developed based on some specific components: a) detection of the four corners of the zig-zag pattern, b) detection of the laser dot, and c) extraction of the variables Acuity and Speed from the coordinates of the laser dot for each video. A video paradigm of the process can be viewed in [28] of the whole process as presented in Fig. 2.

### Detection of the four corners

**Fig. 3:**
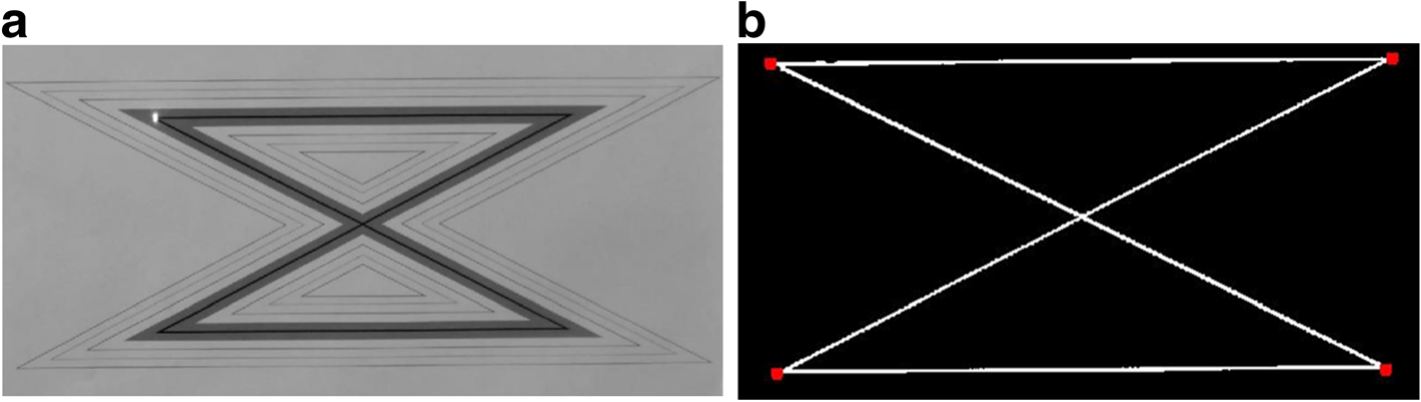
The detection of the four corners of the zig-zag pattern. **a** The initial image, and **b** the four corners detected by the method and depicted as red squares

An important step of our method was the calculation of the distance of the laser dot from the corners of the zig-zag pattern and in order to achieve this an image binarization algorithm was utilized. First the image was transformed to grayscale [29] and then thresholded in order to detect the black line that lies in the green area of the zig-zag pattern. Then using this binary image, the four more distant on-pixels 1) up left, 2) up right, 3) down left and 4) down right were detected as shown in Fig. 3 below. The four corners were detected only once, since the zig-zag pattern paper was stably fixed to a wall.

### Detection of the laser dot

The next step was to detect the laser dot area that was projected from the laser on the paper of the zig-zag pattern. This is an image segmentation problem and a number of algorithms are available for that kind of problems [30]. Our choice was to create a method that will be able to track the laser dot with high segmentation accuracy and as quick as possible, since the videos that we had to process included a large number of images e.g. a 44 s video included: 44(sec) × 30(frames/s) = 1320 images. In order to make this feasible we chose to binarize the color image and then clear the thresholded image from various artefacts [31]. This allowed to achieve the detection of the laser dot in an average computation time of 30msecs.

### Extraction of variables

**Fig. 4:**
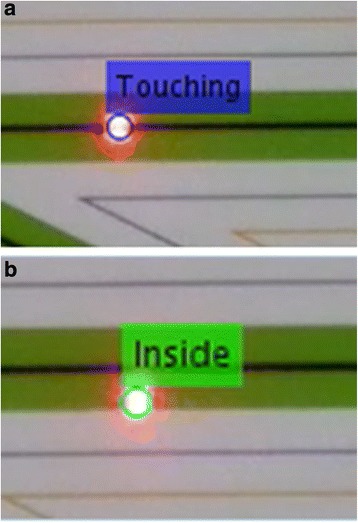
Two cases of the laser dot: (**a**) touching the black line (Top) and (**b**) being inside the green area but is not touching the black line (Bottom)

**Fig. 5:**
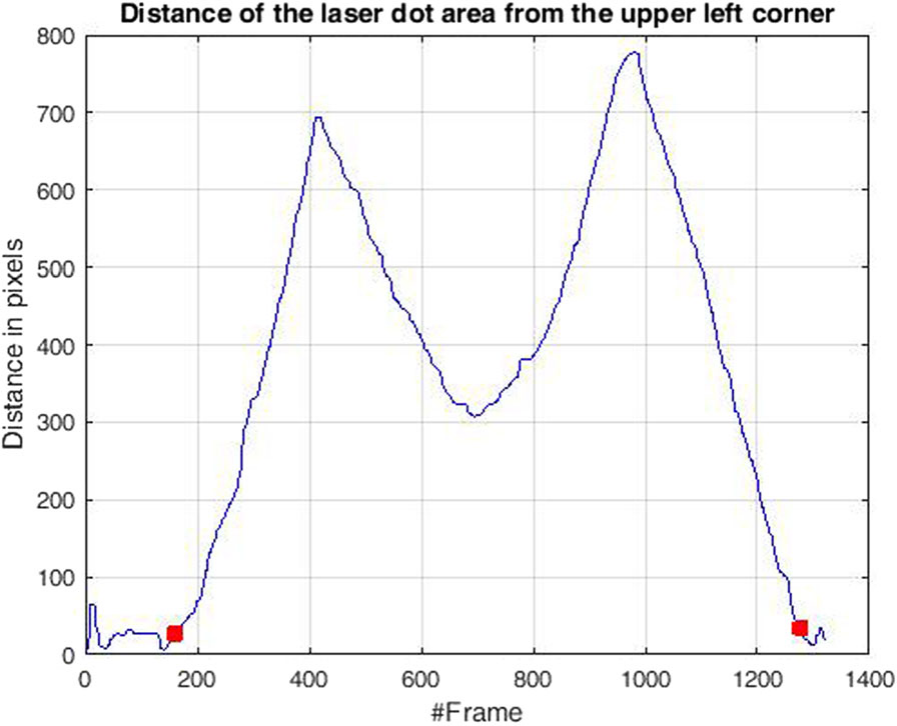
Detection of the Start and End frame (red squares) using the distance of the laser dot from the upper left corner

In the proposed framework, initially we detected whether the laser dot area was touching the black line or not (Fig. 4) with the next step to detect the start and stop frames of the trial. Detecting the start and stop was done using the distance of the laser dot from one corner of the zig zag pattern. An example of the detection of the start and stop time point is depicted in Fig. 5.

The outcome variables from the test included movement acuity (Acuity) calculated as a percentage of the trial time that the laser dot was on the black target line, and the average movement speed (Speed) in mm per second. These outcome variables were calculated for each trial and each hand and direction. Mean values for the three trials of the same hand and direction were calculated for the analysis of differences between the dominant and non-dominant hand and between movement directions. Movements initiated in a clockwise direction, i.e. from left to right, were for left hand named palmar direction and for right hand dorsal direction, while movements initiated in an anti-clockwise direction, i.e., from right to left, were for left hand named dorsal direction and for right hand palmar direction. Defining wrist movements as palmar and dorsal was done due to biomechanical and physiological factors of the hand that can influence movement behaviour of dominant and non-dominant hand as the sample included both left and right handed participants. Mean values for the three trials with the same hand (left and right hand, respectively) and direction (dorsal and palmar, respectively) were calculated for the analyses of the differences between participants with and without hand pain disorders.

### Statistical analyses

Statistical analyses were performed with IBM SPSS Statistics 23 and Microsoft Excel 10. Histograms and Shaphiro-Wilks tests were used to assess normality of distribution for each variable and parametric or non-parametric analyses were chosen based on whether data were normally distributed or not. All Acuity variables had normal distribution, while some of the Speed variables did not. Paired t-tests were used to analyse differences in test performance regarding Acuity between the dominant and non-dominant hand and between the movement directions initiated in dorsal and palmar direction. Independent t-tests were used to analyse any differences between individuals with, compared to without, a musculoskeletal pain condition involving the hand within the previous 12 months. Independent t-tests were used to analyse differences between women and men, and Pearson’s Correlation Co-efficient for analysing relationship between Acuity and age. Spearman Correlation was used to investigate associations between Acuity and Speed.

Intra Class Correlation (ICC_2.1_) with two-way random consistency single measures was used to evaluate relative intra-session test-retest reliability, and Standard Error of Measurements (SEM) was used to evaluate the expected random error (trial to trial noise in the data) of Acuity between the three trials for each hand and movement direction as a measure of absolute reliability [32]. SEM was calculated by dividing the standard deviation of the difference values (mean difference of difference between trial 1 and 2, and trial 2 and 3) by the square root of 2 [26]. Repeated measure analyses of variance (ANOVA) was used to investigate any differences between the three test trials indicating a systematic bias for each of the four hand movement tests.

## Results

Fifty-three participants agreed to participate of which three were excluded. Two people were excluded because they were diagnosed with multiple sclerosis and one due to major impairment of a hand from a previous injury several years ago. The data presented in this study is thereby from a group of 50 physiotherapists, with or without a hand pain disorder. The participants included 21 females and 29 males, with a mean age of 32 +/− 9.6 years, mean height 176 +/− 9.5 cm and mean weight 74 +/− 12.3 kg. Seven participants were left handed, 42 right handed and one reported being ambidextrous. None of the participants had a fractured lower arm, hand or finger within the previous 12 months. Six participants reported some kind of reduced hand functioning at the day of testing, related to weakness, reduced range of motion and/or pain. Ten participants reported having experienced left hand pain within the previous 12 months, and nine participants had experienced right hand pain, of these five reported both left and right hand pain.

All 50 participants performed the three trials of each of the four hand movements, i.e., with left and right hand and in both palmar and dorsal direction with each hand, leading to a total of 600 video films to be analysed. Due to technical problems such as movement of the camera during the test or blocked view of part of the target by the participants hair or shoulder there were missing data from 9 trials. Therefore 591 trials were included in the analyses.

Any difference between women and men, and associations between age and performance was analysed with pooled data of movement direction for dominant and non-dominant hand, respectively. No difference was found between women and men regarding acuity, 73.7 and 72.6 (*p* = 0.714) for dominant hand and 68.4 and 68.1 (*p* = 0.718) for non-dominant hand, respectively. There was no significant correlation between age and acuity in this group *r* = −0.127 (*p* = 0.379) for dominant hand and *r* = 0.032 (*p* = 0.824) for non-dominant hand.

There was a significant better acuity for dominant hand compared to non-dominant hand among participants. This was seen when initiating the test in both dorsal (i.e., right to left direction for left handed and left to right direction for right handed participants) and palmar direction (i.e., left to right direction for left handed and right to left direction for right handed participants) as shown in Table 1. Speed was significantly faster for dominant hand in the palmar direction but there was no difference in the dorsal direction (Table 1).

**Table 1:** Comparison of Acuity (percentage of time on black line) and Speed (mm/s) mean (standard deviation) values between the dominant and the non-dominant hand in dorsal and palmar movement directions

Dominant vs nondominant hand movements	Acuity %	*p*-value	95% CI
Dominant hand in dorsal direction	72.7 (10.3)	< 0.001	3.3–8.6
Non-dominant hand in dorsal direction	66.8 (10.1)		
Dominant hand in palmar direction	73.6 (11.6)	< 0.001	2.4–6.1
Non-dominant hand in palmar direction	69.3 (10.8)		
	Speed mm/s		
Dominant hand in dorsal direction	54.1 (21.3)	0.966	−4.5–5.7
Non-dominant hand in dorsal direction	54.0 (22.0)		
Dominant hand in palmar direction	57.0 (21.8)	0.044	0.1–6.7
Non-dominant hand in palmar direction	53.5 (20.6)		

The 95% confidence interval (CI) refers to the difference between dominant and non-dominant hand for the different movement directions

When comparing the movement direction, there was a significantly better acuity in palmar direction of 69.5, compared to dorsal direction, 66.9, (*p* = 0.01) for non-dominant hand, but no significant difference for dominant hand in palmar compared to dorsal direction, 73.9 and 72.7, respectively (*p* = 0.295).

**Fig. 6:**
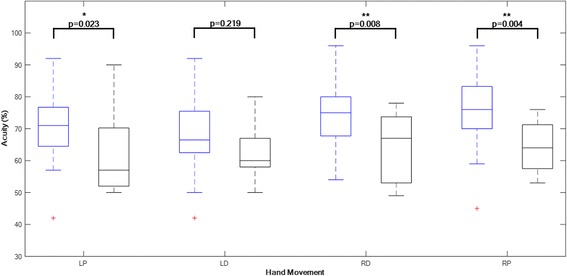
Boxplots of Acuity data (percentage of time on black line) for participants without right hand pain (blue boxes) and those with right hand pain (black boxes) within previous 12 months. Comparisons are made for each hand movement: left hand palmar (LP), left hand dorsal (LD), right hand dorsal (RD) and right hand palmar (RP). Box plots show median and 25st and 75rd percentiles, and T-bars representing minimum and maximum values. The points (+) represent outliers that have values deviating more than 1.5 times the box height from the median values

Participants with, compared to without, right hand pain within last 12 months had a significantly reduced acuity for right hand motion in both dorsal and palmar direction (*p* < 0.01), but also for left hand in palmar direction (*p* < 0.05) as presented in Fig. 6.

**Fig. 7:**
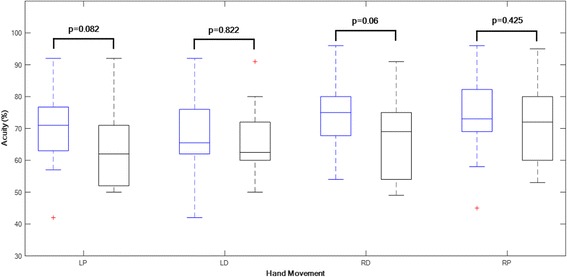
Boxplots of Acuity data (percentage of time on black line) for participants without left hand pain (blue boxes) and those with left hand pain (black boxes) within previous 12 months. Comparisons are made for each hand movement: left hand palmar (LP), left hand dorsal (LD), right hand dorsal (RD) and right hand palmar (RP). Box plots show median and 25st and 75rd percentiles, and T-bars representing minimum and maximum values. The points (+) represent outliers that have values deviating more than 1.5 times the box height from the median values

There was, however, no significant difference between participants with, compared to without, left hand pain within the last 12 months for any of the left, or right, hand motion in either palmar or dorsal (*p* > 0.05), data presented in Fig. 7.

Within the whole group of participants, there was a significant negative correlation between Acuity and Speed for each hand and movement direction (*p* < 0.01), as presented in Table 2.

**Table 2:** Spearman rank correlation analyses of Acuity and Speed. Comparisons were made for each hand movement

Left and right hand movement variables	rho	*p*-value
Acuity RD^a^	−0.718	< 0.001
Speed RD^a^		
Acuity RP^b^	−0.596	< 0.001
Speed RP^b^		
Acuity LP^c^	−0.653	< 0.001
Speed LP^c^		
Acuity LD^d^	−0.646	< 0.001
Speed LD^d^		

^a^Right hand Dorsal direction; ^b^Right hand Palmar direction; ^c^Left hand Palmar direction; ^d^Left hand Dorsal direction

Assessments of the intra-session repeatability of the three trials of each motion test shows ICC values between 0.68 and 0.81 and SEM values ranging between 5.0-6.3 for Acuity, see Table 3.

**Table 3:** Repeatability of Acuity (percentage of time on black line) of the three trials of each hand movement

Dominant and non-dominant hand movements	Trial 1	Trial 2	Trial 3	ICC^a^	SEM^b^	F-value	*p*-value
Acuity dominant hand in dorsal direction	71.6 (12.4)	73.9 (11.3)	72.6 (11.0)	0.69 (0.56-0.80)	6.1 (5.3–7.4)	1.67	0.194
Acuity dominant hand in palmar direction	71.6 (13.4)	73.9 (12.5)	74.8 (11.6)	0,81 (0.71-0.88)	5.3 (4.6–6.3)	4.27	0.017
Acuity non-dominant hand in dorsal direction	63.7 (10.9)	68.6 (11.5)	68.5 (11.8)	0.68 (0.55-0.79)	6.2 (5.4–7.4)	4.40	0.015
Acuity non-dominant hand in palmar direction	68.0 (11.5)	71.1 (10.7)	70.3 (11.5)	0.79 (0.69-87)	4.9 (4.2–5.9)	9.61	< 0.001

Data are presented as mean (standard deviation) values. ICC_2.1_ and SEM are presented together with their 95% confidence intervals. F- values and *P*-values from repeated measures analyses of the three trials of each hand movements for evaluation of any systematic bias

^a^Intra Class Correlation; ^b^Standard Error of Measurement

There was a systematic bias with a learning effect, i.e., improved acuity, over the three trials of each movement direction except for dominant hand in dorsal direction, see Table 3.

## Discussion

The objective of this study was to develop and conduct an initial validation of a novel test for sensorimotor function, or more specifically, movement sense of the hand. The presented results show that there was a significantly better acuity for the dominant hand as well as a reduced acuity for participants with right hand pain within the last 12 months. Moreover the findings prove that there was a clear negative correlation between Acuity and Speed indicating a speed-accuracy trade off commonly found in manual tasks. The repeatability of the test showed acceptable ICC values (0.68-0.81) and SEM values ranging between 5.0-6.3 for Acuity.

The usability of the laser pointer combined with motion quantification by image analysis methods to 2D video recordings has been investigated in a previous study were the laser technique was compared to a 3D electromagnetic tracking system [33]. Their results showed high correlation in the time and frequency domains between the two methods, which gives support to accurately capture movement behavior with laser pointer and image analyses. The zig-zag pattern was chosen in the study to assess straight line movement acuity in horizontal and diagonal directions which are included in various functional movements of the hand, e.g., horizontal pointing movements. The diagonal movement from the upper lateral corner to the lower medial corner involves a movement from radial extension to ulnar flexion of the wrist, also referred to as the “dart throwing motion” (DTM) described in many functional tasks [34]. The symmetry of the pattern allows for assessment in both palmar and dorsal directions with both left and right hand which facilitates assessment of hand independent of side of injury and for comparisons between hands.

To date no previous study has, to the authors’ best knowledge, developed a clinical test with a laser pointer combined with automatic scoring of movement acuity. Due to the immense importance of the sensorimotor function of the hand in daily activities and the lack of objective clinical assessment in hand rehabilitation this study focus on filling this gap by development and preliminary evaluation of the validity of a clinical test for movement sense of the wrist. Several results from this initial study indicate support for the validity of the test. Firstly, performance was significantly better for dominant compared to non-dominant hand. Improved motor skills of dominant hand have been reported in several studies involving goal-directed movements to visual targets [35–37], and has been associated with physiological factors including improved visual feedback processing and enlarged hand representation in motor cortex at the contralateral hemisphere [35, 38]. Secondly, a common finding in hand movement tasks, and also seen in this study, is the typical speed-accuracy trade off presented as a negative correlation between speed and acuity. This means that increased acuity is performed with reduced movement speed, while increased speed leads to reduced acuity. This general finding in hand movement tasks was reported by Woodworth already at the end of 19th century [39] and was further investigated by Paul Fitts in a series of experiments [40] and is sometimes referred to as Fitts’ law. This has since then been confirmed in other studies [41, 42]. Thirdly, in this convenience sample of 50 physiotherapists the test revealed significantly reduced acuity among participants who had experienced right hand pain within the previous 12 months compared to those who had not. Although not significant, there was a trend also for reduced acuity among participants experiencing left hand pain within the previous 12 months. This is in line with previous studies reporting decreased proprioception in musculoskeletal disorders [10–12] and indicates that the test has potential as a clinical assessment of sensorimotor function of the hand by evaluation of the movement sense of the wrist.

The validity of the test was moreover supported by the use of blinded test leader and automatic scoring of outcome variables. The software program was developed for automatic objective analyses of the video recordings for Acuity and Speed scores for each separate trial. Each trial was also visually evaluated by the software programmer for validation of outcome scores calculated by the software. Both software programmer and test leader were blinded regarding participants handedness and whether reporting pain or not within previous 12 months.

The repeatability of Acuity for the three test trials showed acceptable relative reliability as shown by the ICC values, > 0,6. Also, the absolute reliability was relatively good shown by SEM values between 0.05–0.63 which are < 10% of the mean values for all hand movements. Three out of the four hand movements showed a significant improvement over the trials indicating a learning effect. This systematic bias needs to be considered if using the test repeatedly, e.g., for evaluation of treatment effects. Further knowledge about the reproducibility of the test is needed, for example by using a test-retest design where participants are assessed with a longer time interval between test sessions and inter-rater reliability assessment by two or more assessors.

A previous review of motion tracking systems for rehabilitation concludes that current systems are generally technologically complex and often space demanding [22]. To be useful in the clinical setting there are some issues that need to be considered, such as cost, size, weight, function, operation and automation, with a design that preferably allows for wireless real time operation, easy manipulation, user-friendly graphical interface, accurate measures and portability [22]. The feasibility of the presented method in this current study was supported by the fact that data were collected at two different locations by the same test-leader with affordable and easy to use equipment available at any common clinical setting including a hand held laser pointer, a target pattern printed on a A3 paper taped to a wall, a chair with back and armrests, a 1.00 m measure for exact distance between laser pointer to target and a DV camera mounted on a tripod. All 12 trials, including instructions and test trials took approximately 15 min to complete per person. The data were calculated after the test with a new automatized software program for analyses of the video recordings of the test. Future development is planned to involve online calculation of the outcome variables to further improve clinical feasibility.

### Clinical implications

The method described and evaluated in this study has the potential to provide clinicians with a feasible and affordable objective assessment tool for movement sense of the wrist, and thereby fill a current gap in the clinical setting. The test can be used to identify individuals who are expected to gain from sensorimotor training and for evaluation of treatment effects. This method has applications also for other target patterns and tests using the laser pointer, as well as for other joints and body parts. In wrist joint instability disorders, e.g., the DTM has been recommended as a functional and important task to include in assessment and rehabilitation as it is involved in many daily activities [34]. Part of the zig-zag pattern involves the DTM, as described above, and this specific sequence can be analysed exclusively. The DTM and similar functional movements can also be assessed with specifically designed target patterns with the laser pointer technique, including target pattern for performance of the joint position sense test, which is currently often assessed with a goniometer [20]. Regarding other body parts, has a similar test as presented in the current study been used for assessment of neck motions were a laser pointer was attached on the head [43]. In the neck study was, however, the movement behavior assessed with an electromagnetic tracking system which is not needed with the method presented here. Further development of target patterns and software program will allow for objective automatic scoring in assessments of various sensorimotor function tests of the hand, neck or other body parts. The laser pointer technique also has potential as a training device for the hand, neck and other body parts [44].

### Study limitations and recommendations for future research

This study investigated movement acuity on working age physiotherapists with and without hand pain within previous 12 months. This study does not include data on selected patient groups with specific wrist, hand or finger musculoskeletal disorder, e.g., instability, osteoarthritis or fractures. Moreover there were no neurological disorders included in this study, e.g., stroke, Parkinson’s disease or cerebral palsy. Future studies should, in accordance with the COSMIN tool [45], investigate various patient populations with conditions that can affect sensorimotor function of the hand as well as various professions and age groups. Moreover, future studies should include test-retest reliability designs where the test is repeated with, e.g., a week or longer between the test sessions to increase knowledge about reproducibility over time and reproduced between testers to assess inter-rater reliability. It would also be valuable to evaluate the construct validity of the test by using a 3D motion analysis system to assess the actual movement of the hand, as well as assessment of responsiveness by including the test before and after a specific intervention. It should be mentioned that the test presented here assessed one element of proprioception, i.e., movement sense, and the task is performed against a visual target. This can be considered functional due to the importance of eye-hand coordination in many daily tasks. However, other specific tests of proprioception, including non-visual tests may be relevant to include in research and clinical work, e.g., joint position sense, movement discrimination and force sense tests, since they assess various aspects of proprioception and movement behavior.

## Conclusions

This preliminary study indicates that a test involving a tracking task of a zig-zag pattern with a laser pointer and automatic scoring of acuity from video recordings may be a valid and feasible test for assessment of movement sense of the hand. Further knowledge about its validity should be gained from studies on various patient groups and reliability studies with test-retest and inter-rater designs.

## Abbreviations

ANOVA: Analysis of variance
CNS: Central nervous system
DTM: Dart throwing motion
ICC: Intra class correlation
SEM: Standard error of measurement
